# Measurement of axial vertebral rotation using three-dimensional ultrasound images

**DOI:** 10.1186/1748-7161-10-S2-S7

**Published:** 2015-02-11

**Authors:** Quang N Vo, Edmond HM Lou, Lawrence H Le

**Affiliations:** 1 Department of Biomedical Engineering, University of Alberta, Edmonton, Canada; 2 Department of Surgery, University of Alberta, Edmonton, Canada; 3 Department of Radiology and Diagnostic Imaging, University of Alberta, Edmonton, Canada; 4 Department of Biomedical Engineering, Ho Chi Minh City University of Technology, Ho Chi Minh City, Vietnam

## Abstract

**Background:**

Axial vertebral rotation (AVR) is one of the important parameters to evaluate the severity and predict the progression of scoliosis. However, the AVR measurements on radiographs may underestimate its actual value. This pilot study investigated a new three-dimensional (3D) ultrasound method to measure AVR.

**Methods:**

Three cadaveric vertebrae T7, L1, and L3 were scanned with a 3D medical ultrasound system. Nine sets of ultrasound data, the vertebral rotation from 0 to 40° with 5° increments, were recorded from each vertebra. An in-house program was developed to reconstruct and measure the 3D vertebral images. The rotation of each reconstructed vertebra was determined by the angle between the line going through the centres of either laminae (L-L) or transverse processes (TP-TP) and a reference vertical plane. Three raters measured the rotation in 3 sessions, in which they used the mouse pointer to select the L-L or TP-TP according to their knowledge of vertebral anatomy. The program detected the 3D coordinates of these points and calculated the AVR.

The intra-class correlation coefficients (ICCs) were used to calculate the intra-reliability and inter-reliability. The mean absolute difference (MAD±SD) and the range of difference (RD) between the actual values and the average measurements of each rater were computed to evaluate the accuracy of methods.

**Results:**

When rotation was greater than 30° for both L1 and L3, all raters found it difficult to determine one of the lamina areas due to the lack of ultrasound information in an area behind the spinous process. Therefore, the corresponding measurements were excluded. The ICC values of the intra-reliability (L-L, TP-TP) for the three raters were (0.987, 0.991), (0.989, 0.998) and (0.997, 1.000), respectively; meanwhile, the inter-reliability were 0.991 for (L-L) and 0.992 for (TP-TP). All ICC values were greater than 0.98 indicating both methods were highly reliable. The MAD±SD values (L-L, TP-TP) for the three raters were (1.5±0.3°, 1.2±0.2°), (1.6±0.3°, 1.3±0.3°), and (1.7±0.5°, 0.9±0.2°), respectively. The RD (L-L, TP-TP) were (0-4.5°, 0-3.5°), (0-5.1°, 0-4.3°), and (0-5.1°, 0-2.8°) for the three raters, respectively.

**Conclusions:**

The (L-L) and (TP-TP) methods could be used to measure AVR reliability from the 3D ultrasound images.

## Background

Scoliosis is a complex 3D deformity of spine associated with axial vertebral rotation. Adolescent Idiopathic Scoliosis (AIS) accounts for about 80% of all cases and affects 1.5 – 3% of the population [1]. The Cobb angle and axial vertebral rotation are two major factors which can be used to measure the severity of scoliosis, estimate the risk of progression and evaluate the treatment outcomes. Currently, the Cobb angle is gold standard recommended by the Scoliosis Research Society. However, the AVR measurement is often ignored in scoliosis clinics due to time consumption.

Ultrasound has been proposed as an alternative to X-ray based approaches to monitor and image AIS [2-5]. Spinous processes, laminae, transverse processes can be visualized and used as landmarks to measure the Cobb angle and the AVR [2-5] on the frontal or transverse planes of the ultrasound images.

The objectives of this pilot study were to investigate the reliability and the accuracy of the AVR measurements based on either the centres of laminae or the centres of transverse processes on 3D ultrasound images. The ethical approval for this study was granted by the local health research ethics board.

## Methods

### Materials and experimental setup

**Figure 1:**
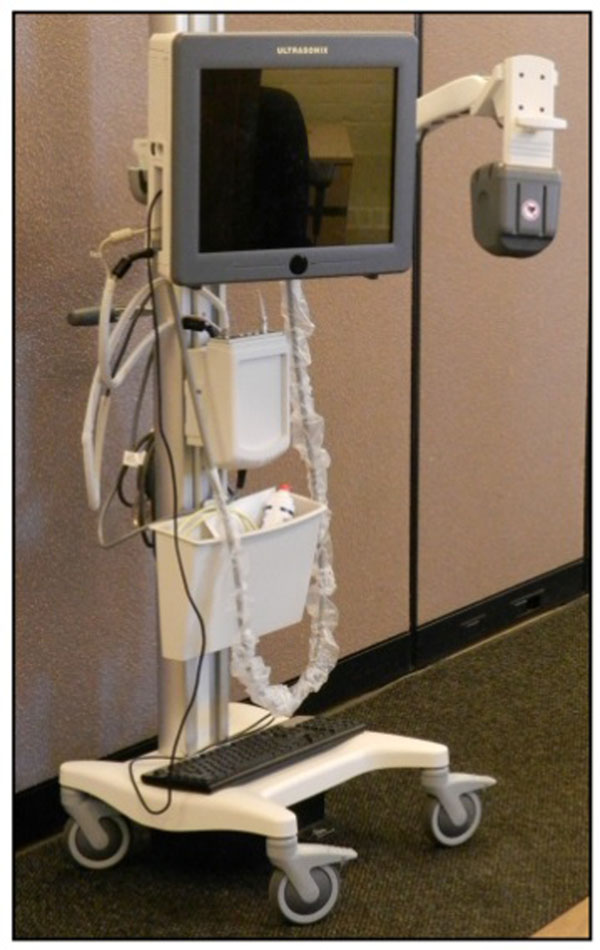
The 3D medical ultrasound machine

The Ultrasonix SonixTablet (Ultrasonix Ltd., Canada), a 3D medical ultrasound system consisting of a magnetic position and orientation tracking system and a 3D Guidance device (driveBAY, Ascension Ltd., USA), was used in this study (Fig. 1). The convex transducer with an active scanning area of 60 mm x 15 mm connected to the ultrasound unit was set to operate at 4.0 MHz.

**Figure 2:**
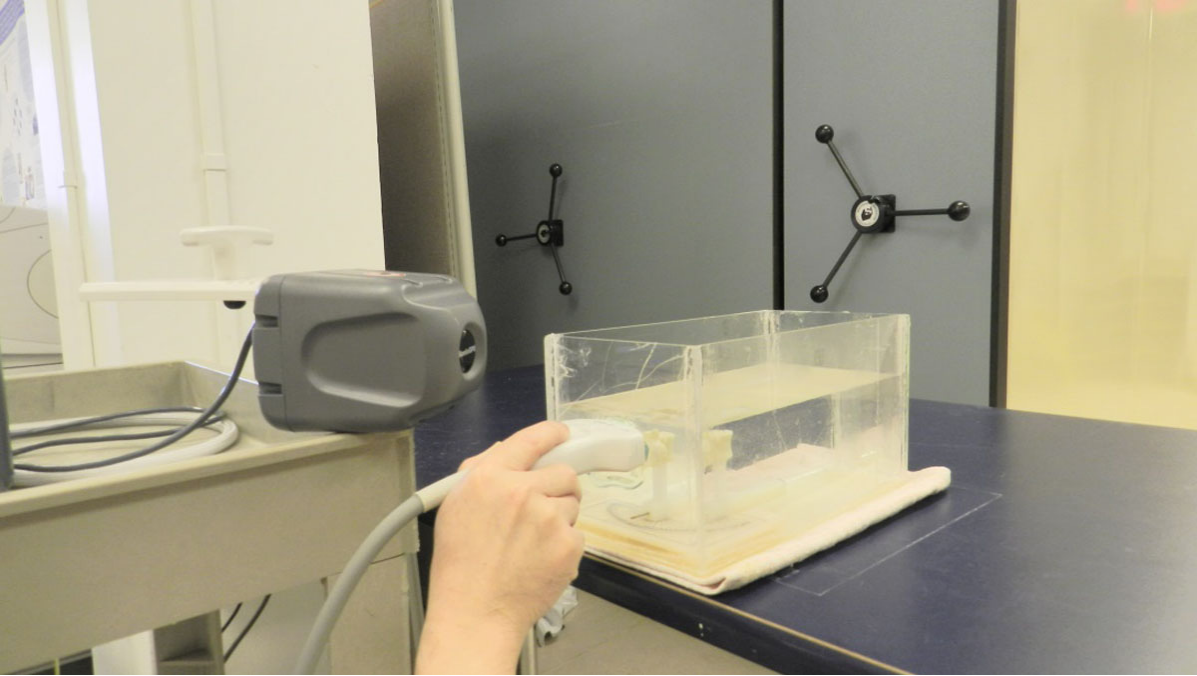
The experimental setup to scan a cadaveric vertebra

Fig. 2 shows a cadaveric vertebra attached to a protractor which is used to indicate the degree of vertebral rotation relative to the scanning wall of the container. The vertebral rotation was adjusted from 0 – 40° with a 5° increment. During the experiment, the tested vertebra was immersed in water to mimic the body setting.

Three cadaveric vertebrae T7, L1, and L3 were scanned to acquire 27 data sets. An in-house developed program was then used to reconstruct and measure the 3D vertebral images.

### The AVR measurement

**Figure 3:**
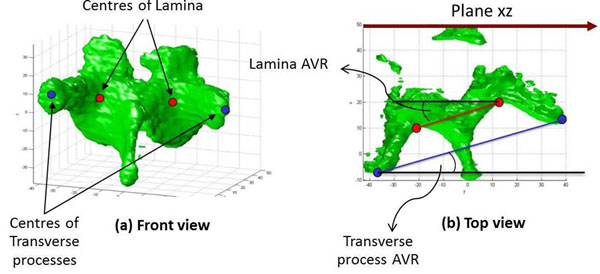
**(a) - The frontal view (left) and (b) - The transverse view (right) of the 3D reconstructed T7 vertebra for axial vertebral rotation measurement** (a) The centres of laminae (red dots) or transverse processes (blue dots) were manually located by using the computer mouse pointer. (b) The rotation of each reconstructed vertebra was automatically determined by the angle between the line going through either the centres of laminae (L-L) or the centres of transverse processes (TP-TP) and a reference vertical plane (xz).

Three raters with 6 months of scoliosis experience participated into this study. To measure AVR, the raters used the computer mouse pointer to manually locate either the centres of laminae or the centres of transverse processes on the 3D reconstructed vertebral images according to their knowledge of vertebral anatomy. The program detected the 3D coordinates of these points and calculated the AVR based on either the centres of laminae or the centres of transverse processes. The rotation of each reconstructed vertebra was automatically determined by the angle between the line going through either the centres of laminae (L-L) or the centres of transverse processes (TP-TP) and a reference vertical plane (the scanning wall), in this case plane xz as shown in Fig. 3.

Three raters who were blinded with the rotation information measured the rotation in 3 sessions in one week apart to minimize memory bias. The intra-class correlation coefficients (ICCs), two-way random and absolute agreement, were used to calculate the intra-reliability and inter-reliability. The accuracy of the measurement method was determined based on the mean absolute difference (MAD ± SD) and the range of difference (RD) between the true values and the average measurements of each rater.

## Results

**Figure 4:**
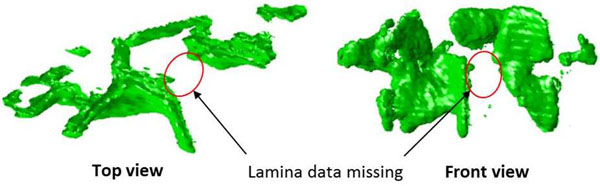
The top view (left) and the frontal view (right) of the 3D reconstructed L3 vertebra for the lamina data missing due to ultrasound blocking.

One of the lamina areas were missing on L1 and L3 when the AVR was greater than 30°. This was because it could not be displayed due to ultrasound blocking of the spinous process (Fig. 4). However, it is not the case for T7. Therefore, a total of four corresponding measurements were excluded out of 27 data sets.

The intra-reliability among three measurements of each rater is shown in Table 1. The mean absolute difference (MAD) and the range of absolute difference (RD) between the mean value measured by each rater and the actual value are also illustrated in Table 2 and Table 3. In addition, the inter-reliabilities among the three raters were 0.991 and 0.992 for the L-L and TP-TP measurements, respectively. The MADs between any two raters are shown in Table 4.

**Table 1:** The intra-reliability among three measurements of each rater.

	Rater 1	Rater 2	Rater 3
L – L	0.987	0.989	0.997

TP - TP	0.991	0.998	1.000

**Table 2:** The mean absolute difference [absolute(mean measured value – actual value)].

	Rater 1	Rater 2	Rater 3
L – L	1.5 ± 0.3°	1.6 ± 0.3°	1.7 ± 0.5°

TP - TP	1.2 ± 0.2°	1.3 ± 0.3°	0.9 ± 0.2°

**Table 3:** The range of absolute difference [absolute(mean measured value – actual value)]

	Rater 1	Rater 2	Rater 3
L – L	0 - 4.5°	0 - 5.1°	0 - 5.1°

TP - TP	0 - 3.5°	0 - 4.3°	0 - 2.8°

**Table 4:** The mean absolute difference between two raters

	Rater 1 vs. Rater 2	Rater 2 vs. Rater 3	Rater 1 vs. Rater 3
L – L	1.06 ± 0.93°	1.18 ± 1.59°	0.93 ± 1.42°

TP - TP	1.34 ± 1.32°	0.94 ± 0.68°	0.71 ± 0.85°

## Discussion

Three different cadaveric vertebrae with different structures were used to investigate the feasibility of measuring the AVR from the 3D reconstructed ultrasound images. The ICC values of the intra-reliability and the inter-reliability were greater than 0.98 indicating both methods (L-L and TP-TP) were highly reliable. The ICC, the MAD and RD values of the TP-TP method are higher or better than the L-L method; however, it does not show a clinical significant difference (3-5^o^ error) on the measurements. Both the L-L and TP-TP methods could measure the AVR reliability from the 3D ultrasound images. In *in-vivo* cases, the L-L method is preferable as the transverse processes may be interfered with ribs. Also, the distance between the laminae is shorter, which is more likely to be covered by the ultrasound signals.

Due to the ultrasound characteristics, the more the vertebra rotates, the higher chance the spinous process blocks the ultrasound signals which may cause missing lamina data.

## Conclusions

The results demonstrated that L-L and TP-TP could be used interchangeably to measure AVR from the 3D ultrasound images. The actual accuracy and rotation measurement limitation may require more investigation in *in-vivo* cases.

This is the extended abstract of IRSSD 2014 program book [6].

## Competing interests

The authors declare that they have no competing interests.

## Authors' contributions

QNV performed the study, programmed to reconstruct 3D images and to measure AVR, carried out the acquisition of ultrasound data and the statistical analysis and interpretation of measurement data, and drafted the manuscript.

EL designed and guided the study and revised the manuscript.

LHL conceived of the study, and participated in its design and coordination and helped to revise the manuscript.

All authors read and approved the final manuscript.

## Declarations

Publication charges for this collection were funded by IRSSD 2014 Sapporo.

This article has been published as part of *Scoliosis* Volume 10 Supplement 2, 2015: Research into Spinal Deformities 9: Short Papers from the IRSSD 2014 Meeting. The full contents of the supplement are available online at http://www.scoliosisjournal.com/supplements/10/S2.
